# RNA-Seq Reveals the mRNAs, miRNAs, and lncRNAs Expression Profile of Knee Joint Synovial Tissue in Osteoarthritis Patients

**DOI:** 10.3390/jcm12041449

**Published:** 2023-02-11

**Authors:** Linghui Qiao, Jun Gu, Yingjie Ni, Jianyue Wu, Dong Zhang, Yanglin Gu

**Affiliations:** 1 Wuxi Xishan People’s Hospital (Wuxi Branch of Zhongda Hospital Affiliated to Southeast University), Wuxi 214000, China; 2 Department of Orthopedics, Jiangnan University Medical Center, Wuxi 214000, China

**Keywords:** osteoarthritis, miRNA, lncRNA, inflammation, synovial tissue

## Abstract

Osteoarthritis (OA) is a chronic disease common in the elderly population and imposes significant health and economic burden. Total joint replacement is the only currently available treatment but does not prevent cartilage degeneration. The molecular mechanism of OA, especially the role of inflammation in disease progression, is incompletely understood. We collected knee joint synovial tissue samples of eight OA patients and two patients with popliteal cysts (controls), measured the expression levels of lncRNAs, miRNAs, and mRNAs in these tissues by RNA-seq, and identified differentially expressed genes (DEGs) and key pathways. In the OA group, 343 mRNAs, 270 lncRNAs, and 247 miRNAs were significantly upregulated, and 232 mRNAs, 109 lncRNAs, and 157 miRNAs were significantly downregulated. mRNAs potentially targeted by lncRNAs were predicted. Nineteen overlapped miRNAs were screened based on our sample data and GSE 143514 data. Pathway enrichment and functional annotation analyses showed that the inflammation-related transcripts *CHST11, ALDH1A2, TREM1, IL-1β, IL-8, CCL5, LIF*, miR-146a-5p, miR-335-5p, lncRNA GAS5, LINC02288, and LOC101928134 were differentially expressed. In this study, inflammation-related DEGs and non-coding RNAs were identified in synovial samples, suggesting that competing endogenous RNAs have a role in OA. TREM1, LIF, miR146-5a, and GAS5 were identified to be OA-related genes and potential regulatory pathways. This research helps elucidate the pathogenesis of OA and identify novel therapeutic targets for this disorder.

## 1. Introduction

Osteoarthritis (OA) is characterized by articular cartilage degeneration, subchondral osteosclerosis, osteophyte formation, and synovitis. OA is the most common chronic joint disease in the elderly [1,2,3]. An estimated 250 million people have knee OA worldwide, and the incidence rate is increasing [4,5]. This disorder has significant health and economic burden, and the current treatment methods cannot effectively prevent disease progression. The pathogenesis of OA is complex and multifactorial. Recent studies have shown an association between synovial tissue and OA.

OA can lead to low-grade synovitis. Increasing evidence shows that synovitis can increase OA symptoms and cartilage degeneration. In OA, the synovium is infiltrated by fibroblast-like synoviocytes and other immune cells [6]. Synovial tissue obtained by closed needle or arthroscopic biopsy can help diagnose OA [7]. Interestingly, the miRNA expression pattern is similar between synovial fluid and synovial tissue [8]. This study evaluated synovial tissue, which is most severely affected by OA.

Epigenetic regulators, including long non-coding RNAs (lncRNAs) and miRNAs, mediate OA [9,10]. LncRNAs are non-coding RNAs (ncRNAs) with more than 200 nucleotides and are widely transcribed in the human genome. LncRNAs are abnormally expressed in human diseases and affect disease progression [11]. For instance, the dysregulated expression of HOTAIR and CIR in OA cartilage indicates that these lncRNAs can assist in OA diagnosis and prognosis and can be used as biomarkers of disease progression [12,13].

miRNAs are small (19–25 base pairs) highly conserved ncRNAs that act as post-transcriptional regulators of gene expression. These molecules bind to target mRNAs to form miRNA-mRNA complexes, leading to target transcript degradation and translation inhibition [14]. A single miRNA can target multiple transcripts, and individual mRNAs can be regulated by several miRNAs, characterizing a complex regulatory network [15,16]. Moreover, some lncRNAs have miRNA-binding sites and act as miRNA sponges in cells, preventing the inhibition of target mRNAs by miRNAs, increasing the expression of target genes, and forming a regulatory network of competing endogenous RNAs (ceRNAs).

Many studies have shown that the expression of miR-140 is low in OA [17,18]. In this respect, the intra-articular injection of miR-140 can alleviate OA progression in rats by regulating extracellular matrix (ECM) homeostasis and can become a treatment option for OA [19]. Furthermore, a new method for treating OA is the use of exosomes to introduce miR-140 into chondrocytes [20]. CircSERPINE2 expression is relatively low in OA tissues, and the injection of CircSERPINE2 alleviates OA in a rabbit model. CircSERPINE2 regulates apoptosis and ECM metabolism in human chondrocytes (HCs) by targeting miR-1271-5p and ERG [21]. Similarly, circRNA.33186, acting as a sponge of miR-127-5p, is directly targeted by MMP-13 in an OA mouse model [22].

This study analyzes the patterns of expression of lncRNAs, miRNAs, and related mRNAs in the knee joint synovial tissue of 8 OA patients, and compared the expression profiles with two controls. Then we download the miRNAs expression data from the Gene Expression Omnibus (GEO) database (GSE143514) to identify the overlapped differentially expressed miRNAs (DEM). GO and KEGG functional enrichment analyses were performed to elucidate molecular functions and pathways in OA. In enriched pathways, inflammatory pathways were focused, and related differentially expressed genes (DEGs) were further analyzed based on the GSE114007 dataset. Severe inflammation was found in the synovial tissue and was associated with pain and structural decline [23]. Our study analyzes the patterns of expression of lncRNAs, miRNAs, and mRNAs in OA synovial tissues and predicts inflammation-related target genes.

## 2. Methods and Materials

### 2.1. Patients and Samples

Synovial tissue samples were obtained during joint surgeries performed in the Wuxi No. 2 People’s Hospital in 2021 and were snap-frozen in liquid nitrogen. All patients provided written informed consent and were treated according to the ethical guidelines of the research ethics committee of our hospital. Transcriptome analysis was performed in eight OA patients and two patients with the popliteal cyst. The diagnosis was performed according to the 2010 ACR/EULAR criteria. All OA patients were end-stage lesions requiring surgical treatment, and there were no significant differences in the extent of the lesions. Eight biopsies from OA patients served as the intervention group, and two biopsies from patients with popliteal cysts patients served as controls. Since intact synovial tissue in healthy individuals is difficult to obtain in practice, patients with simple popliteal cysts were selected for this study. It was determined that none of these patients had any symptoms or history of OA or knee injury, and the possibility of having OA was completely excluded from the imaging examination. The relevant blood parameters also excluded OA, joint cavity infection, and rheumatoid arthritis in this group of patients. Therefore, the synovial tissues of these two patients with simple popliteal cysts excluded the possibility of various inflammatory changes and could be considered healthy synovial tissues. This was used as a control group to carry out the study. We had access to information that could identify individual participants during or after data collection.

### 2.2. RNA Library Construction and Sequencing

Total RNA was isolated and purified using TRIzol (Invitrogen, Carlsbad, CA, USA) following the manufacturer’s instructions. The amount and purity of RNA were determined using a NanoDrop ND-1000 spectrophotometer (NanoDrop, Wilmington, DE, USA). RNA integrity was assessed using an Agilent 2100 Bioanalyzer, and samples with an RNA integrity number >7.0 were considered to be high-quality samples. Approximately 5 mg of total RNA was used to deplete ribosomal RNA according to the recommendations of the Ribo-Zero™ rRNA Removal Kit (Illumina, San Diego, CA, USA). rRNA-depleted RNA was fragmented using divalent cations under high temperatures. First-strand cDNA was synthesized using reverse transcriptase and random hexamers, and second-strand cDNA was synthesized in a buffer containing polymerase, followed by end-repair, poly(A)-tailing, and sequencing adaptor ligation.

Paired-end adapters were ligated to the DNA fragments, and size selection was performed with AMPure XP beads. After UDG treatment of second-strand DNA, the ligated products were amplified by PCR using the following amplification conditions: initial denaturation at 95 °C for 3 min, eight cycles at 98 °C for 15 s, 60 °C for 15 s, and 72 °C for 30 s, and an extension step at 72 °C for 5 min. The amplification products were purified using AMPure XP beads, resuspended in the elution buffer, and heat-denatured. The average read size of the cDNA library was 300 ± 50 bp. Paired-end sequencing was performed on an Illumina HiSeq 4000 platform (LC Bio, Hangzhou, China) following the manufacturer’s recommendations.

### 2.3. Bioinformatics Analysis

Raw reads were processed using proprietary software ACGT101-miR (LC Sciences, Houston, TX, USA) to remove adapter dimers, reads with low-quality bases, abundant RNA (rRNA, tRNA, snRNA, and snoRNA), and PCR duplicates. Unique sequences with a length of 18–26 nucleotides were mapped to human reference genome HG19 using BLAST and miRBase 22.0 (miRBase database, http://www.mirbase.org/, 26 January 2022). Unique sequences that mapped to mature miRNAs in hairpin arms were considered known miRNAs, and unique sequences that mapped to the other arm of known precursor hairpin opposite the annotated mature miRNA-containing arm were considered to be novel 5′ or 3′-derived miRNA sequences. The remaining sequences were mapped to other databases using BLAST and miRBase 22.0 and mapped pre-miRNAs were aligned against human databases to determine their genomic locations and were considered known miRNAs. Unmapped sequences were aligned against human genomes, and hairpin RNA structures were predicted from flanking 80 nt sequences using RNAfold software (http://rna.tbi.univie.ac.at/cgi-bin/RNAfold.cgi, 19 February 2022).

### 2.4. Analysis of Differential Expressed miRNAs (DEmiRNAs) and Target Genes Prediction

Differential expression of miRNAs based on normalized deep-sequencing counts was analyzed by selectively using Fisher exact test, Chi-squared 2X2 test, Chi-squared non-test, Student t-test, or ANOVA based on the design of the experiment. The significance threshold was set to be 0.01 and 0.05 in each test.

In order to predict the most abundant miRNA target genes, we used two computational target gene prediction algorithms (Targets can, V5.0 and Miranda, v3.3a) to identify miRNA binding sites. The data predicted by the two algorithms were combined to calculate the overlaps. The GO terms and KEGG Pathway of these most abundant miRNAs, and miRNA targets were also annotated.

### 2.5. LncRNA Transcript Assembly

Adaptor sequences and reads with low-quality bases were removed using Cutadapt. Sequence quality was verified using FastQC (http://www.Bioinformatics.Babraham.ac.Uk/projects/fastqc/, 16 March 2022). High-quality sequences were mapped to the genome of Homo sapiens using Bowtie2 and Hisat2. Mapped reads from each synovial sample were assembled into transcripts using StringTie, and transcripts from all samples were assembled de novo into a transcriptome using Perl scripts. Transcript expression levels were estimated using StringTie and edgeR.

### 2.6. LncRNA Identification, Analysis of DE mRNAs and lncRNAs, and Prediction of Target Genes

The transcripts that overlapped with known mRNAs and transcripts shorter than 200 bp were discarded. Transcripts with coding potential were predicted using CPC and CNCI. Transcripts with CPC scores < −1 and CNCI scores < 0 were removed, and the remaining transcripts were considered lncRNAs.

The relative expression of mRNAs and lncRNAs was measured as fragments per kilobase of exon per million fragments mapped using StringTie. DE mRNAs and lncRNAs (log2 fold change > 1 or <−1 at *p* < 0.05) were identified using edgeR.

DE genes at 100 Kb upstream or downstream of DE lncRNAs were selected as potential cis-target genes using Python scripts. The potential functions of these target genes were determined using BLAST2GO. Statistical significance was established at *p* < 0.05.

## 3. Results

### 3.1. Clinical and Biochemical Features of the Included Individuals

The patient’s details are shown in Table 1. After admission, all tests were completed to exclude the possibility of exogenous infection.

### 3.2. Differentially Expressed mRNA, miRNA and LncRNA Profile

A total of 77821 mRNAs, 890 miRNAs, and 47522 lncRNAs were identified in 10 synovial tissues. in the OA group, we found 343 mRNAs significantly upregulated and 232 mRNAs significantly downregulated compared with the control group. For lncRNAs, 270 were significantly upregulated and 109 were significantly downregulated. For miRNAs, 247 were significantly upregulated and 157 were significantly downregulated (*p* < 0.05, fold change > 2).

**Figure 1:**
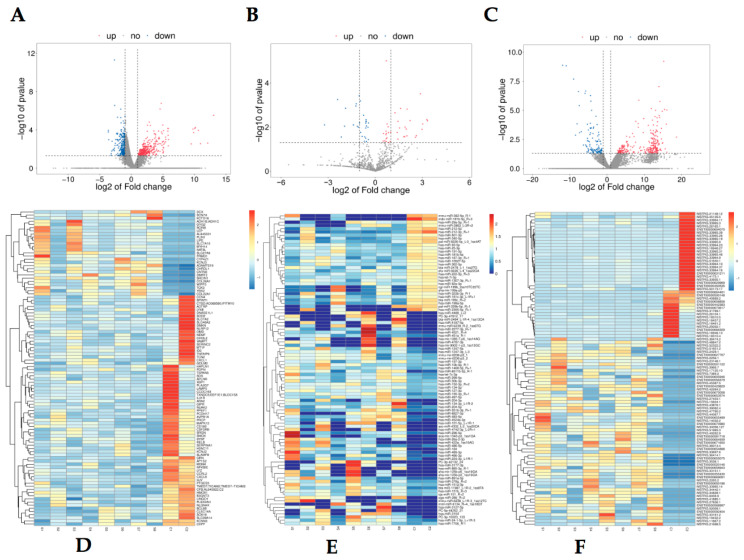
Expression profiles of differentially expressed RNA in OA groups. A total of upregulated mRNAs and downregulated mRNAs are displayed in (**A**) the heat map and (**D**) the volcano plot; upregulated miRNAs and downregulated miRNAs are displayed in (**B**) the heat map and (**E**) the volcano plot; upregulated lncRNAs and downregulated lncRNAs are displayed in (**C**) the heat map and (**F**) the volcano plot; red represents upregulation and blue represents downregulation.

The most upregulated lncRNA was NEAT1 with a 210-fold change; the most downregulated lncRNA was FP671120 with a 0.047-fold change. The most upregulated mRNA was LEP with a 57-fold change; the most downregulated mRNA known was LAMP5 with a 0.079-fold change. Table 2 shows the top five up- and downregulated mRNAs, miRNAs, and LncRNAs. mRNA, miRNA, and LncRNA volcano and heat maps are shown in Figure 1.

**Figure 2:**
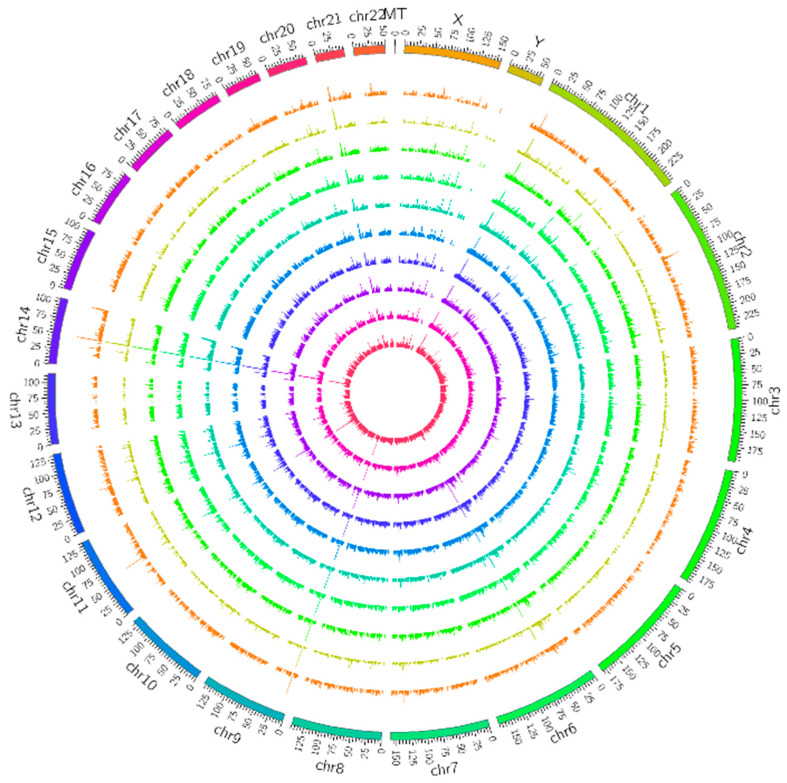
Long noncoding RNAs (lncRNA) mapping shows lncRNA distribution in each chromosome. The outer 8 rings are the OA group, and the inner 2 are the control group.

As shown in Figure 2, the detected lncRNAs were widely distributed on all classes of chromosomes. We used circus (ww.circos.ca, 3 May 2022) software to perform genomic mapping of the lncRNAs obtained from the screening. Mapping was performed by taking each chromosome as a basic unit per 25 MB, respectively, and the expression of lncRNAs in each segment was counted for mapping when visualizing the lncRNA genomes in different samples, and the genomes of different lncRNA types were mapped. The number of lncRNAs in each segment was counted during visualization.

### 3.3. GO and KEGG Enrichment Analysis of lncRNAs/miRNA Target mRNAs

**Figure 3:**
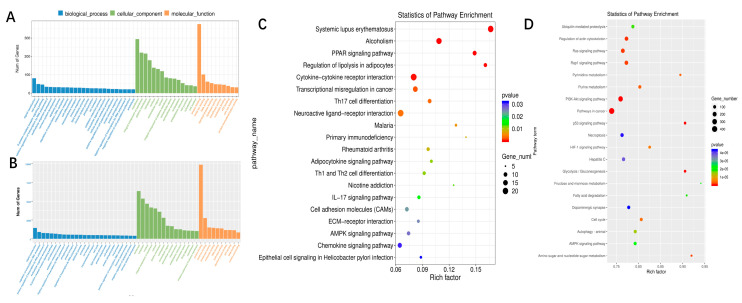
GO and KEGG enrichment analysis. (**A**) Gene ontology (GO) annotations and (**C**) Kyoto Encyclopedia of Genes and Genomes (KEGG) pathway analysis for the mRNAs regulated by lncRNA. (**B**) Gene Ontology (GO) annotations and (**D**) Kyoto Encyclopedia of Genes and Genomes (KEGG) pathway analysis for the miRNAs. The top 20 according to the *p* value of each analysis are displayed in KEGG pathways.

GO analysis was performed on all the target mRNAs expressing significantly different increases. As shown in Figure 3A, negative regulation of receptor-mediated endocytosis, ribosomal large subunit binding, and filopodium membrane signaling pathway is the main functions associated with dysregulated lncRNAs.

We performed KEGG pathway analysis on the target mRNA of this dysregulated lncRNA. The main enrichment pathways include ABC transporters, EGFR tyrosine kinase inhibitor resistance, and allograft rejection (Figure 3C).

We also predicted the possible target genes of differential miRNA. Go enrichment analysis showed that thromboxane-a synthase activity, B cell apoptotic process, and fibrin odium membrane were the main enriched GO terms (Figure 3B). Pathway in cancer was the main enriched KEGG signaling pathway (Figure 3D).

### 3.4. Prediction of the Interaction between Differentially Expressed lncRNA-mRNA

To further study the regulation of lncRNAs on gene expression through a possible ceRNA network during OA, we predicted the interaction between the upstream and downstream of differentially expressed lncRNAs within 100 k bp. A total of 55 pairs of differential lncRNA-mRNA were identified. These differentially expressed lncRNAs may affect the OA process through cis-regulation of mRNAs, which were differentially expressed. The results are shown in Table 3 (TOP10).

### 3.5. Expression Validation of OA-Related Overlapped DEMs, lncRNAs, and mRNAs in Synovial Tissue

We focused on the expression of OA-related genes and inflammatory markers, some of which were dysregulated in OA. OA-related genes *CHST11* and *ALDH1A2* and inflammatory genes *TREM1, IL-1β, IL-8, CCL5*, and *LIF* were dysregulated in OA. Furthermore, miR-146a-5p and miR-335-5p (targeting the *IL-1β* gene) and lncRNAs GAS5 (miR-34a-*Bcl2* axis), LINC02288 (miR-374a-3p-*RTN3* axis), and LOC101928134 (targeting the *IFNA1* gene) were differentially expressed in the synovial tissue of OA patients vs. the control group based on our sample data and GSE143514.

**Figure 4:**
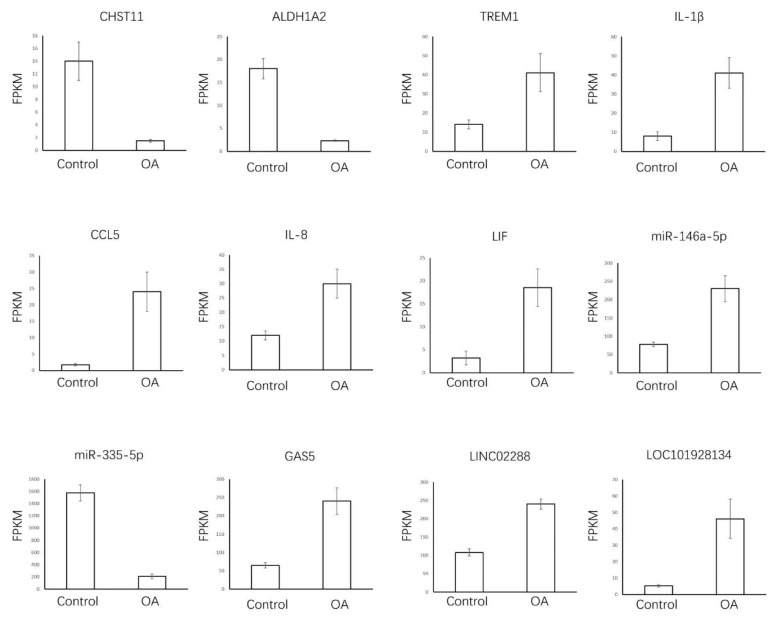
A total of 12 OA-related lncRNAs, miRNA, and mRNAs enriched in synovial tissue samples. We applied independent samples t-test. OA-related genes and inflammatory markers that have been well-studied were selected and the expression of each gene in the OA and control groups was examined separately and bar graphs were plotted.

For upregulated genes, the expression of TREM1, LIF, miR146-5a, and GAS5 increased, which was consistent with our sample data (Figure 4). However, IL-1β, IL-8, CCL5, LINC02288, and LOC101928134 were not significantly different (*p* > 0.05). For downregulated genes, CHST11 and miR335-5p were incompatible with the previous results. Thus, TREM1, LIF, miR146-5a, and GAS5 were identified to be OA-related genes in our study.

## 4. Discussion

OA affects the joints of the knees, hands, hips, and spine and is the main cause of limited mobility among the elderly [24,25]. Although genetic susceptibility, aging, obesity, and joint malalignment are risk factors for OA, the pathogenesis of OA is unclear [26,27]. Therefore, other than total joint replacement, interventions that delay disease progression and cartilage degeneration are currently unavailable [28]. In the past decade, genome-wide association studies have increased the amount of genomic data exponentially. In this study, RNA-seq was used to determine transcriptome changes in the synovial tissue of OA and RA patients. A total of 1358 RNA transcripts were DE in the OA group (860 upregulated and 498 downregulated), and some target genes were involved in inflammation.

Carbohydrate (chondroitin 4) sulfotransferase 11 (CHST11) belongs to the thioltransferase 2 family and catalyzes the transfer of sulfate to position four of the N-acetylgalactosamine (GalNAc) residue of chondroitin. Chondroitin sulfate is the most abundant proteoglycan in cartilage and is widely found on the cell surface and ECM. The abnormal expression of CHST11 is associated with osteochondral dysplasia, short fingers/toes, and other dysfunctions. In 2017, a genome-wide association study found that CHST11 was associated with OA in North American Caucasians with knee OA [29]. In our study, CHST11 was downregulated in the OA group (*p* < 0.05).

The enzyme aldehyde dehydrogenase 1A2 (ALDH1A2) catalyzes the synthesis of retinoic acid from the retinal. Retinoic acid is an active metabolite of vitamin A (retinol), which plays a role in tissue development. Genetic studies in mice have shown that this enzyme, with the help of cytochrome CYP26A1, maintains retinoic acid levels, promotes bone development, and prevents spina bifida [30]. The expression level of ALDH1A2 is closely related to the pathogenesis of hand OA in Icelandic patients [31]. In our study, the expression of ALDH1A2 was significantly downregulated in OA patients.

Toll-like receptor 4 (TLR4) is activated during acute inflammation and increases the expression of TREM1, and the latter triggers the expression of proinflammatory cytokines, including TNF-a and IL-1β [32]. TREM1 is highly expressed in inflamed synovium [33]. Moreover, TREM1 is significantly upregulated in damaged cartilage [34], consistent with our results. The expression of TREM1 in OA patients was higher than that in the RA group, suggesting that the degree of inflammation is increased in OA cartilage. This result was corroborated by the high expression of downstream IL-1β. IL-1β is implicated in many pathological features of OA [35]. IL-1β signaling has been shown to regulate the expression of 909 of 3459 genes in primary human articular chondrocytes, including genes encoding chemokines and inflammatory mediators, such as IL-11 and CCL5 [36]. Other OA-related inflammatory factors include IL-8 and LIF [37].

DE ncRNAs have been identified in synovial samples. miR-146a-5p was upregulated in the articular cartilage and serum of OA patients [38]. miR-335-5p attenuates chondrocyte inflammation in OA by activating autophagy [39]. The overexpression of miR-335-5p downregulates the expression of inflammatory mediators IL-1β, IL-6, and TNF-α and increases the expression of autophagy marker beclin-1 and autophagy-related proteins 5 and 7.

LncRNA GAS5 expression is high in OA cartilage and increases with disease progression. GAS5 acts as a negative regulator of miR-21 and thereby controls cell survival [40]. Similarly, LINC02288 expression was significantly upregulated in a rat model of OA. LINC02288 knockdown reduced IL-1β-induced apoptosis and proinflammatory cytokine production in OA chondrocytes [41]. The lncRNA LOC101928134 was highly expressed in the synovial tissue of OA rats, regulated *IFNA1* expression, and inhibited the JAK/STAT signaling pathway. Moreover, the downregulation of LOC101928134 improved synovial inflammation and cartilage injury, enhanced synovial cell apoptosis, and inhibited chondrocyte apoptosis in OA rats [42].

NEAT1 was the most upregulated lncRNA in the synovial tissue in this study. Despite being significantly downregulated in chondrocytes, NEAT1 controls chondrocyte proliferation and apoptosis through the miR-543/PLA2G4A axis [43] and regulates cartilage matrix degradation through the miR-193a-3p/SOX5 axis [44]. However, NEAT1 is upregulated in synovial cells and is believed to control their proliferation by competitively binding to miR-181c [45]. The distinct expression of NEAT1 in chondrocytes and synovial cells may indicate different pathophysiological mechanisms in OA.

The most upregulated gene in our study, LEP, a weighted gene, was associated with OA and can be a new biomarker and drug target [46]. Studies have shown that plasma LEP levels are positively correlated with the risk of OA [47]. In OA patients, obesity was significantly associated with an increase in LEP promoter methylation. Lysosomal-associated membrane protein LAMP5 is involved in gastric cancer and leukemia. However, no studies have assessed the role of LAMP5 in OA.

While this study focuses on joint-related inflammatory RNAs, other studies have focused on exploring the pathogenesis of OA at the RNA level. Huang ZY et al., used paired scRNA-seq and bulk RNA-seq data to create and validate an “arthritis” specific feature matrix that significantly outperformed the default feature matrix for synovial cells. RNA-seq data for rheumatoid arthritis (RA) and OA were analyzed by estimating relative subsets of RNA transcripts for cell type identification using machine learning tools. The authors, [48] X Zhang et al., retrieved and downloaded mRNA expression data from a comprehensive gene expression database to identify differentially expressed genes (DEGs) in OA and normal individual synovial tissue [49]. Dysregulated genes including USP46, CPVL, FKBP5, FOSL2, GADD45B, PTGS1, ZNF423, ADAMTS1, and TFAM were found to be potentially involved in the pathology of OA. This overlaps with some of the mRNA loci identified in this study. Microarray and RNA-seq data from the comprehensive gene expression database were also obtained for the study by Le Kang et al., who collected joint fluid from patients who underwent inpatient arthroplasty for validation experiments [50]. Ultimately, it was found that there was a difference in gene expression between OA and RA and that the presence of ADAMDEC1 in the joint fluid was a good biomarker for RA. In this study, complete whole genome sequencing was performed and many differentially expressed RNAs were obtained, but in contrast to the above study, no cross-sectional comparisons were made using gene sets from online databases, and no other clinical specimens were collected for validation experiments. This part will be refined in a later study.

The samples for this study were collected largely within three months and then sequenced uniformly on the Illumina platform. This avoids the errors associated with split experiments. If more samples are to be included it may take too long and the long storage time of the samples may lead to loss of RNA fragments. With new sequencing technologies such as long read length, RNA sequencing and advances in direct RNA sequencing (dRNA-seq), more complete data can be obtained to some extent. However, this also means increased costs and no great advantage in sequencing miRNA, mRNA, etc. Moreover, because of the strict entry criteria for this study, there were relatively few patients with simple popliteal cysts without arthritis, which led to a reduction in the number of controls. If conditions permit at a later stage, a multi-center collaboration to increase the sample size and update the technique can be considered for further study.

In conclusion, DE lncRNAs, mRNAs, and miRNAs were identified in the synovial tissue of patients with OA by RNA-seq. Although the ceRNA network was not analyzed in this study, 12 differentially expressed inflammation-related transcripts were identified in the knee joint synovial tissue, suggesting that ceRNAs participate in OA. These data help elucidate the mechanisms underlying OA and develop novel therapeutic targets.

## Figures and Tables

**Table 1:** Clinical characteristics of study population.

	Gender	H/W (cm)/(kg)	Age (Year)	Course (Month)	ESR (mm/h)	CRP (mg/L)
OA1	F	155/51	69	120	14.3	4.7
OA2	F	162/58	71	60	9.2	<0.5
OA3	M	169/71	75	96	3.5	<0.5
OA4	F	157/55	70	72	13.8	8.8
OA5	M	162/64	68	96	19.7	5.1
OA6	M	171/73	71	36	18.7	6.4
OA7	F	163/62	73	48	24.1	1.3
OA8	F	157/53	62	72	18.5	10.5
cyst1	F	159/52	65	5	16.0	3.2
cyst2	M	170/67	68	11	22.6	1.1

**Table 2:** Top 5 significantly up- and downregulated mRNAs, miRNAs and lncRNAs.

Gene Name	log2 (Fold Change)	Regulation	RNA Sort
LAMP5	−3.65	Down	mRNA
HJV	−3.64	Down	mRNA
CXCL8	−2.67	Down	mRNA
DMKN	−2.62	Down	mRNA
PLA2G7	−2.36	Down	mRNA
NAT8L	5.09	Up	mRNA
ADH1B	5.44	Up	mRNA
CYP4Z1	5.78	Up	mRNA
AL845331	5.78	Up	mRNA
LEP	5.83	Up	mRNA
hsa-miR-129-5p	−4.75	down	miRNA
hsa-miR-323b-3p_R-1	−3.23	down	miRNA
hsa-miR-212-3p_R+1	−3.22	down	miRNA
hsa-miR-130b-3p	−3.07	down	miRNA
hsa-miR-21-3p_R+1	−2.93	down	miRNA
hsa-miR-34c-3p_1ss16GA	4.21	up	miRNA
hsa-miR-335-5p_R-1	4.30	up	miRNA
hsa-miR-877-3p_R+1	4.47	up	miRNA
hsa-miR-34c-5p	5.01	up	miRNA
hsa-miR-34b-3p_L-1R+1	6.98	up	miRNA
ARRDC3-AS1	−3.88	Down	lncRNA
GUSBP11	−3.88	Down	lncRNA
MIR4435-2HG	−4.02	Down	lncRNA
CKMT2-AS1	−4.32	Down	lncRNA
FP671120	−4.39	Down	lncRNA
NEAT1	7.72	Up	lncRNA
AP000757	6.86	Up	lncRNA
XIST	6.84	Up	lncRNA
AC060780	6.35	Up	lncRNA
AC008691	5.45	Up	lncRNA

**Table 3:** Top 10 pairs of differential lncRNA-mRNA identified in OA.

mRNA	lncRNA	cis Location	*p* Value
SLX1A	SLX1A-SULT1A3	1 K	−0.70
BTK	ARMCX4	100 K	−0.57
SYNPO2	SEC24D	100 K	−0.50
NBR1	AC060780	10 K	−0.42
REV3L	MFSD4B	100 K	−0.35
CSNK1A1	CARMN	100 K	−0.34
ZFP62	LINC00847	100 K	−0.31
CRIP2	MIR8071-1	100 K	−0.27
FKBP15	FAM225A	100 K	−0.23
DDIT3	MBD6	100 K	−0.22

## Data Availability

The datasets used or analyzed during the current study are available from the corresponding author on reasonable request.

## Abbreviations

ACR/EULAR	American College of Rheumatol/European League Against Rheumatism
ALDH1A2	aldehyde dehydrogenase 1A2
ceRNAs	competing endogenous RNAs
CHST11	Carbohydrate (chondroitin 4) sulfotransferase 11
DE	differentially expressed
DEGs	differentially expressed genes
DEMs	differentially expressed miRNAs
GalNAc	N-acetylgalactosamine
GO	Gene Ontology
lncRNAs	long non-coding RNAs
KEGG	Kyoto Encyclopedia of Genes and Genomes
ncRNAs	non-coding RNAs
OA	Osteoarthritis
TLR4	Toll-like receptor 4
TREM1	Triggering Receptor Expressed on Monocytes 1
